# Supplementary material for: Cold-Induced Nuclear Import of CBF4 Regulates Freezing Tolerance
Source: Int J Mol Sci. 2022 Sep 27;23(19):11417. doi: 10.3390/ijms231911417 (PMC9570231; doi:10.3390/ijms231911417)
Supplement: Supplementary file 1 [file ijms-23-11417-s001.zip › ijms-1906649-supplementary.pptx]

## Slide 1
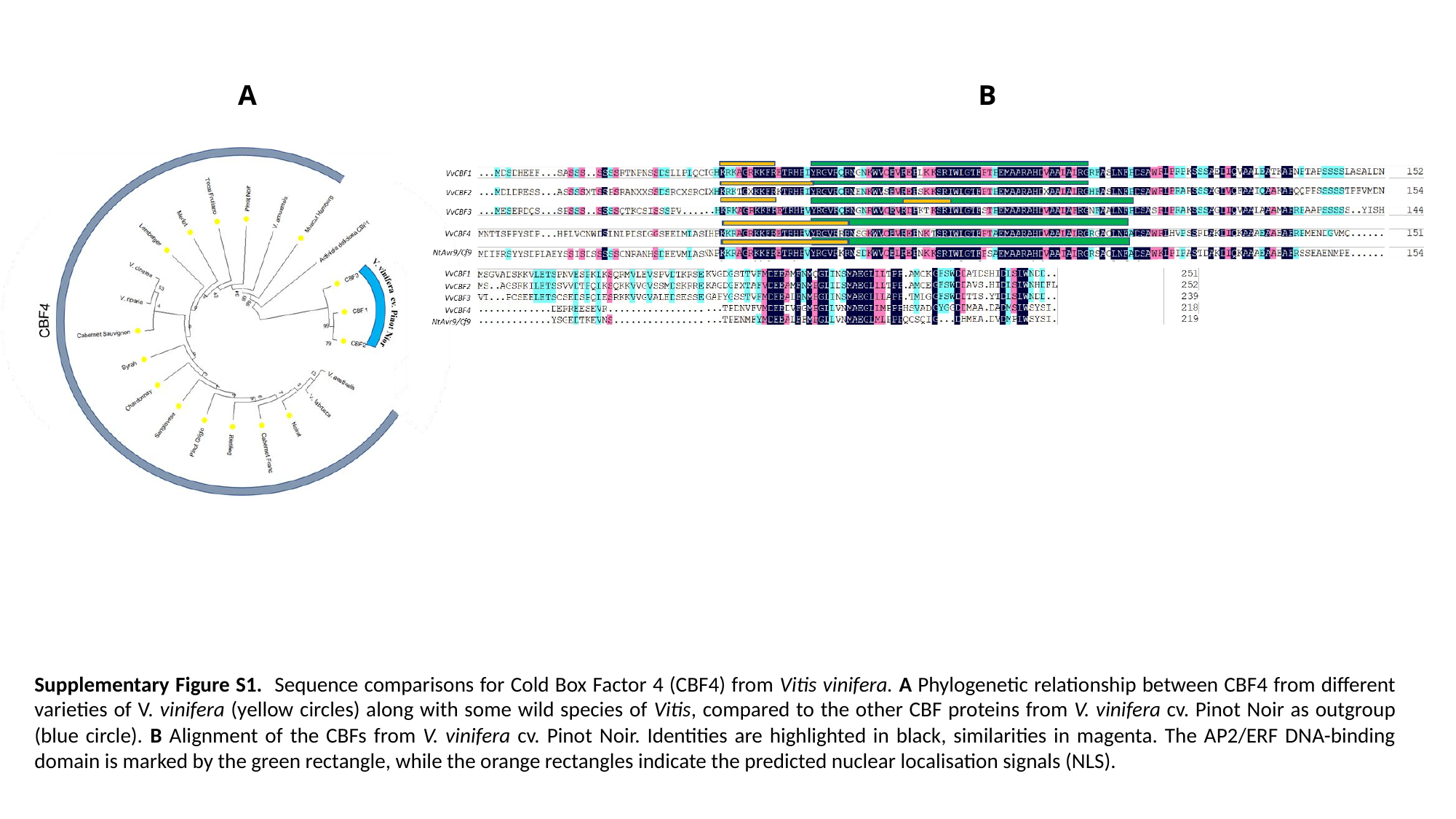

A
B
Supplementary Figure S1. Sequence comparisons for Cold Box Factor 4 (CBF4) from Vitis vinifera. A Phylogenetic relationship between CBF4 from different varieties of V. vinifera (yellow circles) along with some wild species of Vitis, compared to the other CBF proteins from V. vinifera cv. Pinot Noir as outgroup (blue circle). B Alignment of the CBFs from V. vinifera cv. Pinot Noir. Identities are highlighted in black, similarities in magenta. The AP2/ERF DNA-binding domain is marked by the green rectangle, while the orange rectangles indicate the predicted nuclear localisation signals (NLS).

## Slide 2
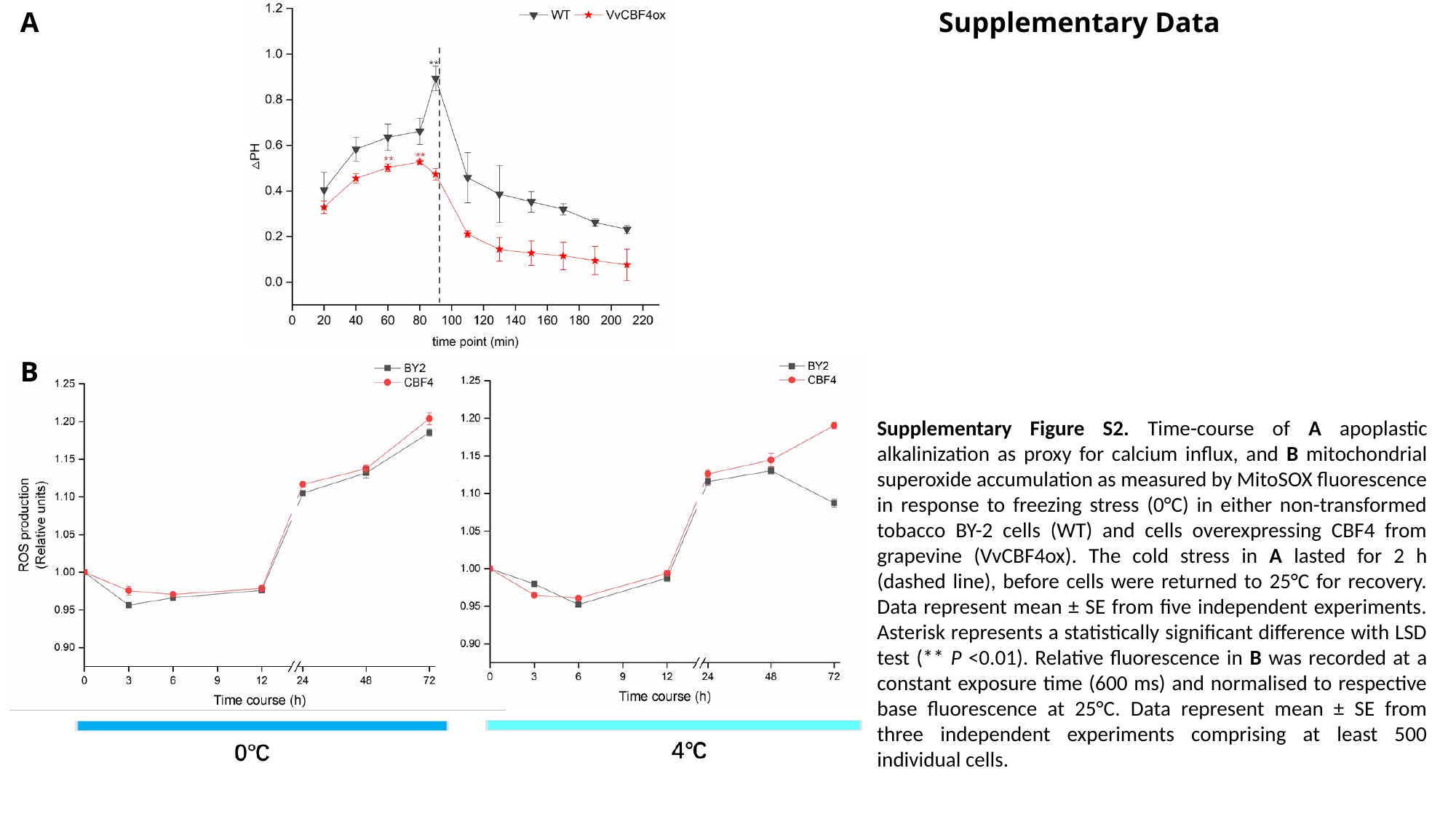

A
Supplementary Data
B
Supplementary Figure S2. Time-course of A apoplastic alkalinization as proxy for calcium influx, and B mitochondrial superoxide accumulation as measured by MitoSOX fluorescence in response to freezing stress (0°C) in either non-transformed tobacco BY-2 cells (WT) and cells overexpressing CBF4 from grapevine (VvCBF4ox). The cold stress in A lasted for 2 h (dashed line), before cells were returned to 25°C for recovery. Data represent mean ± SE from five independent experiments. Asterisk represents a statistically significant difference with LSD test (** P <0.01). Relative fluorescence in B was recorded at a constant exposure time (600 ms) and normalised to respective base fluorescence at 25°C. Data represent mean ± SE from three independent experiments comprising at least 500 individual cells.

## Slide 3
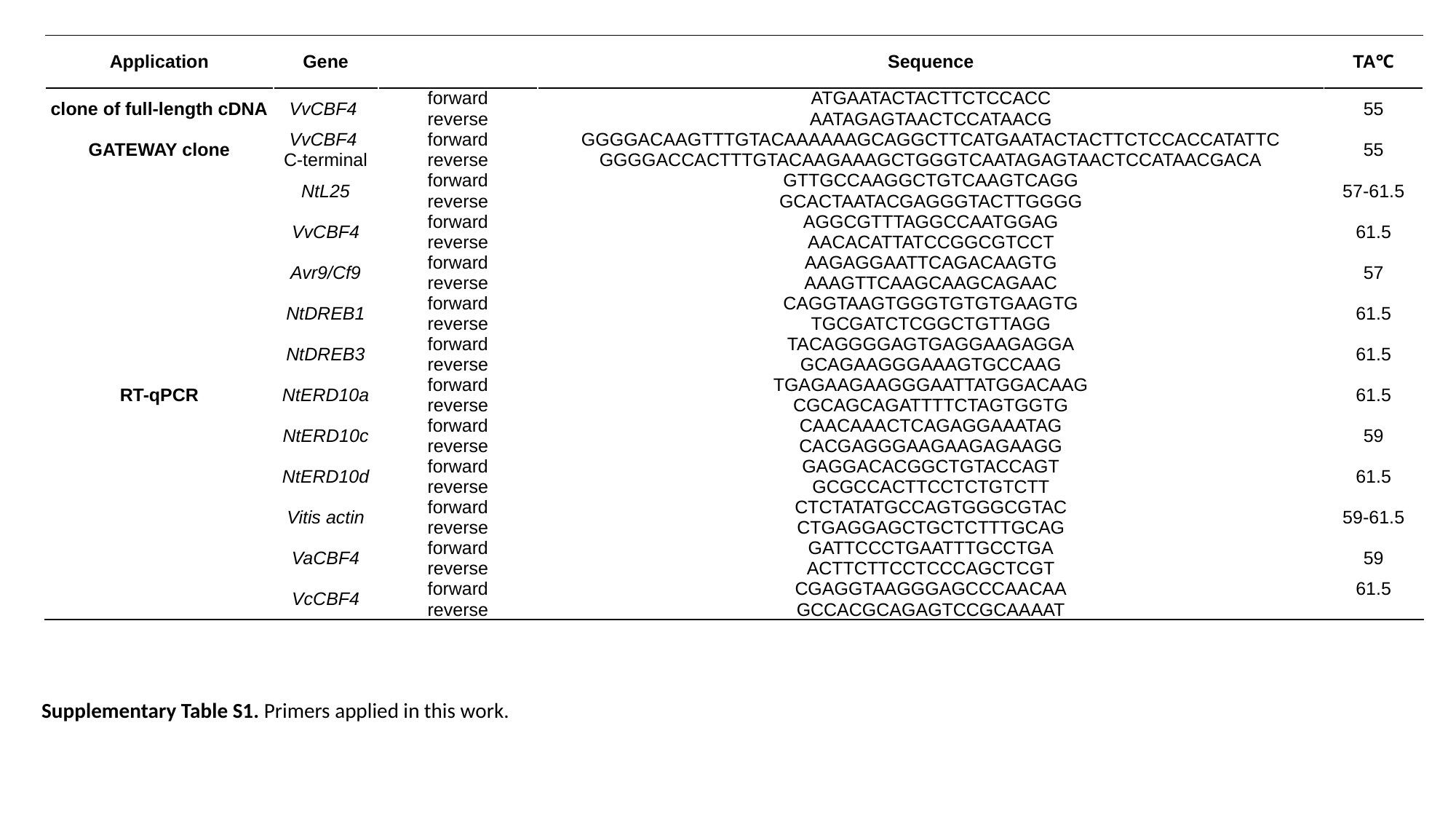

| Application | Gene | | Sequence | TA℃ |
| --- | --- | --- | --- | --- |
| clone of full-length cDNA | VvCBF4 | forward | ATGAATACTACTTCTCCACC | 55 |
| | | reverse | AATAGAGTAACTCCATAACG | |
| GATEWAY clone | VvCBF4 C-terminal | forward | GGGGACAAGTTTGTACAAAAAAGCAGGCTTCATGAATACTACTTCTCCACCATATTC | 55 |
| | | reverse | GGGGACCACTTTGTACAAGAAAGCTGGGTCAATAGAGTAACTCCATAACGACA | |
| RT-qPCR | NtL25 | forward | GTTGCCAAGGCTGTCAAGTCAGG | 57-61.5 |
| | | reverse | GCACTAATACGAGGGTACTTGGGG | |
| | VvCBF4 | forward | AGGCGTTTAGGCCAATGGAG | 61.5 |
| | | reverse | AACACATTATCCGGCGTCCT | |
| | Avr9/Cf9 | forward | AAGAGGAATTCAGACAAGTG | 57 |
| | | reverse | AAAGTTCAAGCAAGCAGAAC | |
| | NtDREB1 | forward | CAGGTAAGTGGGTGTGTGAAGTG | 61.5 |
| | | reverse | TGCGATCTCGGCTGTTAGG | |
| | NtDREB3 | forward | TACAGGGGAGTGAGGAAGAGGA | 61.5 |
| | | reverse | GCAGAAGGGAAAGTGCCAAG | |
| | NtERD10a | forward | TGAGAAGAAGGGAATTATGGACAAG | 61.5 |
| | | reverse | CGCAGCAGATTTTCTAGTGGTG | |
| | NtERD10c | forward | CAACAAACTCAGAGGAAATAG | 59 |
| | | reverse | CACGAGGGAAGAAGAGAAGG | |
| | NtERD10d | forward | GAGGACACGGCTGTACCAGT | 61.5 |
| | | reverse | GCGCCACTTCCTCTGTCTT | |
| | Vitis actin | forward | CTCTATATGCCAGTGGGCGTAC | 59-61.5 |
| | | reverse | CTGAGGAGCTGCTCTTTGCAG | |
| | VaCBF4 | forward | GATTCCCTGAATTTGCCTGA | 59 |
| | | reverse | ACTTCTTCCTCCCAGCTCGT | |
| | VcCBF4 | forward | CGAGGTAAGGGAGCCCAACAA | 61.5 |
| | | reverse | GCCACGCAGAGTCCGCAAAAT | |
Supplementary Table S1. Primers applied in this work.
